# Iodine deficiency in Israeli pregnant women – a time for action

**DOI:** 10.1186/s13584-020-00382-5

**Published:** 2020-05-01

**Authors:** John H. Lazarus

**Affiliations:** 1 Clinical Endocrinology, Thyroid Research Group, School of Medicine, Cardiff University, Cardiff, UK; 2 Regional Coordinator West and Central Europe, Iodine Global Network, Cardiff, UK

**Keywords:** Iodine, Pregnancy, Desalination, Neurodevelopment, Supplement, Fortification public health, Public health, Policy

## Abstract

Iodine is an essential micronutrient for thyroid gland function. Iodine deficiency disorders are a spectrum of conditions affecting the fetus, child, adolescent and adult. Iodine requirements are increased in pregnancy in order that the fetus receives enough maternal thyroxine via transplacental passage. Previous studies in Israel have shown widespread iodine deficiency by measurement of urinary iodine concentrations in school age children and adults. The present study clearly shows iodine deficiency in a group of 2nd trimester women as judged by measurement of serum thyroglobulin (a recently evaluated marker of iodine deficiency). An additional factor in this work is that the subjects all resided in an area using desalinated water. Desalination has previously been shown to significantly reduce the iodine content of water compared to water in Israel not subject to this process.

The data in this group of pregnant women should serve as a wakeup call to the public health community to correct this deficiency which is known to significantly affect child neurodevelopment. There are several issues to be addressed. The knowledge base relating to iodine nutrition especially during pregnancy is low. An educational plan is required. The strategy for achieving adequate iodine nutrition in the population and particularly before and during pregnancy requires urgent review. While iodine supplementation before and during gestation can correct iodine deficiency, the provision of iodised salt in the community is recommended, similar to more than 80% of countries in the world.

It is indeed a time for action to ensure the adequate intellectual performance of Israel’s children.

## Background

Iodine is an essential micronutrient to ensure adequate functioning of the thyroid gland and iodine deficiency (ID) has been recognised for more than a century as an important public health problem [1, 2]. Originally seen mainly in high altitude areas such as the Andes, Himalayas and the Alps it presented as goitre together with cretinism in extreme cases. Administration of iodine as iodine tablets, iodised oil and later as universal salt iodization ameliorated these conditions. In the latter half of twentieth century WHO, UNICEF and The Iodine Global Network recognised that there was a continuing problem and noted the wide clinical spectrum of ID disorders affecting the fetus, neonate child and adult [3]. Non pregnant adults require 150mcg Iodine/day but the requirement rises in pregnancy to 220-250mcg/day. Thyroxine (T4) traverses the placenta and iodine deficiency will reduce the amount of T4 supply to the fetus. Adequate amounts of T4 are required to convert to T3 (triiodothyronine) in the brain in order for developmental processes such as myelination and neuronal migration to occur in a defined temporal pattern. The decrement in brain function in human studies has been evidenced by reduction in school performance in 8 year old children [4, 5] although there is no increase in goitre prevalence. These data have led to the therapeutic suggestion of iodine supplementation in countries where ID is still prevalent in pregnancy usually because there is no sustainable program of universal salt iodization (USI). Today > 80% of countries in the world have iodised salt availability mostly mandatory [6]. However, there are countries, especially in Europe, which have adequate iodine nutrition shown by measurement of urinary iodine concentrations (UIC) in children but where pregnant women have low UIC.

### The Israel situation

In Israel iodine deficiency was not considered to be a problem perhaps because of the proximity to The Mediterranean. While some studies of goitre were performed in the early 60s iodine deficiency was not reported in school age children and pregnant women on a national scale till 2017 [7]. A possible contribution to ID in Israel is the use of desalinated water in certain populations reliant on this type of water supply [8]. A previous study had drawn attention to the fact that mixing desalinated water with groundwater in Israel may contribute to ID. Currently there is no mandatory requirement for iodized salt in Israel although this has been recommended more than 10 years ago [9].

A recent IJHPR article by Rosen and colleagues [10] has evaluated iodine status in 134 pregnant women living in an area reliant on desalinated iodine-diluted water. While iodine concentration in water from Ashkelon was 35 μg/L the water iodine concentrations in several municipalities in the Ashkelon subdistrict ranged between 0 and 9 μg/L. The results showed firstly that the estimated iodine intake by food frequency questionnaire was 187 +/− SD 106 μg/day, significantly below the WHO recommended intake in pregnancy of 250 μg/day. Urinary iodine concentrations were not measured in this study; instead, serum thyroglobulin (Tg) was estimated in all patients. Studies have shown that if the thyroid is ‘stressed’, for example by iodine deficiency there will be an increase in Tg release from the gland. Elevation of Tg levels have correlated with the presence of iodine deficiency [11]. In this study 67% of Tg levels were more than the cut off of 13 μg/L. When serum Tg was examined in relation to estimated dietary iodine intake those women with an I intake of > 220 μg/day 56% still had Tg > 13 μg/L but 74% of women whose I intake was < 220 μg/day had Tg > 13 μg/L, a significant difference. Using the same dietary cut offs the difference in those with Tg > 40 μg/L was more marked (5% vs 21%). These data indicate that there is not a perfect correlation of Tg with iodine status and despite the fact that UIC measurement only relates to short term iodine intake it is unfortunate that this marker was not measured as well. Notwithstanding the difficulty of accurately assessing iodine deficiency it does appear that this population of pregnant women is iodine deficient. It is noteworthy that nearly ¾ of them were not taking any iodine supplements despite the fact that guidance recommending iodine supplementation was officially issued by the Israel MOH Maternal and Child Health on Dec. 13th 2017, following the publication of the national survey [7].

### Future action

What are the implications of this important study for iodine nutrition in Israeli pregnant women? This study adds to the evidence base that iodine deficiency is widespread in Israel in school age children, adults and now pregnant women, particularly so in residents using desalinated water. The government and public health authorities should recognise this and to address this emergency they should implement a programme of universal salt iodization. This programme is not undertaken lightly as it involves interaction with public health personnel, endocrinologists, food industry, salt manufacturers and many government agencies. In addition, it is essential to institute a monitoring system which can feed back results to government and the community at large. This strategy has been shown to be cost effective [12] and would be highly feasible in Israel with its centralized salt industry and proven capacity to produce the iodized salt that is used voluntarily at this time.

This study also raises the role of iodine supplementation before and during gestation. It is apparent that at least in those women who were ingesting more than 220 μg/day of iodine there was a high use of iodine containing supplements. While supplement use can also be cost effective [13], it is probably less cost-effective than USI and may perpetuate health disparities, as supplement use before pregnancy is typically practiced by a minority of women and preferentially by those of higher socioeconomic and educational status.

Another strategic action is to promote awareness in the population about iodine deficiency and its adverse effect on children’s IQ and subsequent school performance.

## Conclusions

The data presented in this paper adds to the evidence base that iodine nutrition in Israel is deficient, particularly in pregnancy. At least half of pregnant women are lacking in a vital nutrient which will result in adverse effects on child neurocognitive performance. The optimum solution to this problem is the introduction of universal salt iodization. This programme should be implemented by government with buy in from relevant stakeholders and be subject to appropriate monitoring and accountability. International agencies including the IGN stand ready to lend their experience to these efforts.

## Data Availability

Not Applicable.

## Abbreviations

I: Iodine
ID: Iodine Deficiency
IQ: Intelligence Quotient
Tg: Thyroglobulin
UIC`: `Urinary Iodine Concentration
UNICEF: United Nations Children’s Fund
USI: Universal Salt Iodization
WHO: World health Association
